# Low awareness of oral and injectable PrEP among high-risk adolescent girls and young women in Kampala, Uganda

**DOI:** 10.1186/s12879-022-07398-z

**Published:** 2022-05-16

**Authors:** Jane Frances Lunkuse, Onesmus Kamacooko, Vincent Muturi-Kioi, Kundai Chinyenze, Monica O. Kuteesa, Matt A. Price, Yunia Mayanja

**Affiliations:** 1 Medical Research Council/Uganda Virus Research Institute and London School of Hygiene and Tropical Medicine (MRC/UVRI & LSHTM) Uganda Research Unit, Plot 51-59 Nakiwogo Road, P.O. Box 49, Entebbe, 256 Uganda; 2 IAVI, 11th Floor, Muthangari Drive, Nairobbi, Kenya; 3 IAVI, 125 Broad St, New York, NY 10004 USA; 4 Department of Epidemiology and Biostatistics, University of California San Francisco, 550 16th Street, San Francisco, CA 94143 USA

**Keywords:** Adolescents, Pre-exposure prophylaxis, Awareness, HIV prevention, Uganda

## Abstract

**Background:**

Adolescent girls and young women (AGYW) account for a disproportionate number of new HIV infections worldwide. HIV prevalence among young sex workers in Uganda is 22.5%. Although pre-exposure prophylaxis (PrEP) is a highly effective biomedical HIV prevention method, awareness of PrEP among AGYW in Uganda has not been studied systematically. We aimed to assess awareness of PrEP and factors associated with awareness of PrEP among AGYW who frequently reported paid sex.

**Methods:**

We conducted a cross-sectional study among 14–24-year old AGYW at high risk of HIV infection in Kampala, Uganda from January to October 2019. Participants were screened for PrEP eligibility using a national screening tool of whom 82.3% were eligible. Data on socio-demographics, behavioral and sexual risks were collected by interview. Awareness of oral or injectable PrEP, the latter of which is currently in late-stage trials, was defined as whether an individual had heard about PrEP as an HIV prevention method. Multivariable robust poisson regression model was used to assess factors associated with oral PrEP awareness.

**Results:**

We enrolled 285 participants of whom 39.3% were under 20 years old, 54.7% had completed secondary education, 68.8% had multiple sex partners in the past 3 months, 8.8% were screened as high risk drinkers’/ alcohol dependent (AUDIT tool) and 21.0% reported sex work as main occupation. Only 23.2% were aware of oral PrEP and 3.9% had heard about injectable PrEP. The prevalence of oral PrEP awareness was significantly higher among volunteers screened as alcohol dependents (aPR 1.89, 95% CI 1.08–3.29) and those with multiple sexual partners (aPR 1.84, 95% CI 1.01–3.35), but was lower among those who reported consistent condom use with recent sexual partners (aPR 0.58, 95% CI 0.37–0.91).

**Conclusions:**

Majority of AGYW were not aware of any kind of PrEP. Those with higher risk behavior, i.e. alcohol dependents or multiple sexual partners, were more aware of oral PrEP. Interventions to increase awareness among female youth are needed. Improving PrEP awareness is critical to increasing PrEP uptake among high-risk AGYW in Uganda.

## Background

Although various HIV prevention interventions (biomedical, behavioral and structural) have mitigated the spread of HIV [1], the number of new cases among adolescent girls and young women (AGYW) remains unacceptably high [2, 3]. In 2019, an estimated 1.7 million adolescents aged 10–19 years were living with HIV across the globe of whom 10% were newly infected [4]. In sub-saharan Africa, AGYW account for 24% of new HIV infections [5] with an estimated HIV prevalence four times higher than their male peers [4, 5]. Additionally, about 5,000 AGYW aged 15–24 years are newly infected with HIV every week [5, 6]. In Eastern and Southern Africa alone, AGYW accounted for 26% of new infections in 2019 [5]. In Uganda, the prevalence of HIV among adolescent sex workers is 22.5% [7]. Most of these are involved in high-risk sexual behavior including sex work and having multiple partners, which has accelerated the spread of HIV in this age group.

The World Health Organization (WHO) recommends the use of oral pre-exposure prophylaxis (PrEP) to reduce the number of new HIV infections among high-risk populations [8], and many national programs have adopted this biomedical HIV prevention strategy [9]. Recent long-acting injectable PrEP findings have shown that it is safe and highly effective [10]. Additionally there many products in the pipeline that are being evaluated for example the PrEP Implant [11]. It is essential that awareness of these methods are built early at the population level. In 2018, of the 3 million people at substantial risk of HIV [12], only 381,580 people were taking oral PrEP worldwide. Of these, only 27% were from sub-saharan Africa with a majority of these PrEP users being AGYW [13]. By July 2020, an estimated 31,000–32,000 people were using PrEP in Uganda, which was lower than the Ministry of Health target of 90,000 people at risk [14].

PrEP is highly effective when adherence is good [15, 16]. A sub study among partners in East Africa, found that high PrEP adherence (> 80%) was associated with 100% PrEP efficacy [17]. However, there is limited awareness of oral PrEP as an HIV prevention method among populations at high risk for HIV in sub-Saharan Africa despite years of science and nascent programs to provide it [18]. The prevalence of PrEP awareness among AGYW remains unknown within Uganda [19] and poor uptake has been reported [18, 20]. Awareness of novel HIV prevention methods among key populations remains important as it will likely motivate uptake and adherence to future prevention products. We therefore need to improve understanding of awareness of both available and future HIV prevention methods. Literature suggests that most individuals at risk support PrEP usage once provided with details on its use and efficacy [21]. Additionally a study among adolescents in the US reported that many persons at risk do not know how to access oral PrEP even if they are aware that they need it [22]. A recent study among AGYW in South Africa found that only 19% were aware of PrEP, and family support and discussing HIV and sexually transmitted infections with sexual partners were associated factors for PrEP awareness [2]. Also, a recent study among Black and Latinx adolescents in the US residing in higher prevalence areas revealed that only 38% knew about PrEP [23].

In recent clinical trials, injectable PrEP has proven to be 89% more effective than oral PrEP [10] and will likely be available as a prevention tool soon. Raising awareness of oral or injectable PrEP is a first and necessary step to increase their use among at-risk AGYW, yet data is still limited in Uganda. Since 2015, the Determined Resilient Empowered AIDS-free Mentored and Safe women (DREAMS) project was implementing a package of HIV prevention services including PrEP provision to AGYW in selected districts of Uganda [24–26]. Also, at the time we enrolled the cohort of AGYW, the Ministry of Health in Uganda was conducting trainings for health workers and rolling out oral PrEP to selected key populations and, 3 clinical trial sites in Uganda were enrolling for the HIV Prevention Trials Network (HPTN084) trial to compare efficacy of long acting injectable cabotegravir to daily oral Truvda [10, 27]. The aim of our study was to determine awareness of oral and injectable PrEP, and to assess factors associated with awareness of oral PrEP among high-risk AGYW in Kampala, Uganda.

## Methods

### Study design and setting

We conducted a cross-sectional analysis of PrEP awareness and associated factors measured from baseline data for a two-year cohort study. Between January and October 2019, 14–24-year-old AGYW were recruited from commercial hotspots (i.e., where sex work is prevalent) and slums of southern and northern Kampala, and invited to enroll into a cohort of AGYW at the Good Health for Women Project (GHWP) clinic located in a peri-urban community in southern Kampala [28]**.** The clinic offered HIV prevention and care services, free general health care and reproductive health services to eligible high-risk women including female sex workers (FSWs).

### Study population and sampling design

The national risk screening tool (evaluates risk in the last 6 months) given by the Ministry of Health (MoH) was used to assess if participants were at high risk for HIV: (i) Being sexually active with at least one of the following i.e. condomless sex with multiple partners, sexually transmitted infections (STIs), and use of post-exposure prophylaxis (PEP); (ii) sharing injections; (iii) and having an HIV positive sexual partner who is not on effective antiretroviral therapy (ART). PrEP Eligibility was then determined by having a positive HIV test and a positive response to any of the risk factors in the MoH risk screening tool.

Eligible Participants were then enrolled into the parent cohort study that was assessing knowledge and preferences of biomedical HIV prevention methods, and uptake of oral PrEP among at-risk 14-to-24-year-old females in Kampala. AGYW were enrolled in the cohort if they were HIV negative, Hepatitis B immune (through prior infection or vaccination) or willing to be vaccinated if susceptible, sexually active in the past 3 months, willing to use effective contraceptive methods, undergo regular HCT, pregnancy testing, and screening for sexually transmitted infections (STIs). Participants were excluded if pregnant or planning pregnancy in the next 12 months; had known allergy to components of oral PrEP drugs, contraceptives or Hepatitis B vaccine; and reported or were diagnosed with an illness that would affect participation in the study.

### Data collection

Trained research assistants collected data using standardized paper questionnaires administered face-to-face. We collected data on socio-demographic and risk behavior (i.e.: number of sexual partners; transactional, anal, group or forced sex; condom use with sexual partners; knowledge of sexual partners’ HIV status; and having STIs). Data were double entered into an Open Clinica database by trained data entrants. Discrepancies were resolved by referring back to the source documents.

### Study variables

#### Primary outcome

Awareness of Oral PrEP was the primary outcome in this study and was measured as a binary variable (Yes/No). As a secondary outcome, awareness of injectable PrEP (a hypothetical product still in clinical trials at the time of this study) was also measured as a binary variable (Yes/No).

Volunteers were also assessed on; (1) their knowledge about of oral and injectable PrEP (Pills were demonstrated using samples at site), (2) the primary source of information for those who reported prior knowledge about these methods and (3) willingness to use these methods after explaining their benefits and frequency of use.

Independent variables included the following: socio-demographics factors included age, highest level of education (below or above primary level), marital status (married, separated, widowed, or single) and main occupation (sex work, no job, other).

Behavioural risk factors included alcohol consumption risk level and drug use. Drug use was defined as use of any illicit drugs in the past 1 month. Alcohol consumption risk level was based on the score of the Alcohol use disorders identification test (AUDIT) questionnaire [29]. Participants were categorised as low risk (AUDIT score < 8), moderate risk/hazardous (AUDIT score 8–19), and high risk/ dependent (AUDIT score ≥ 20).

Sexual risk factors included age at first sex, number of sexual partners in the past 3 months, consistent condom use with sexual partners in the past 3 months, transactional sex (receiving and/ or giving money and goods for sex), history of STI symptoms (abnormal discharge and/ or genital ulcer), frequent traveling away from home in the past 3 months and current contraceptive use.

### Statistical analysis

We analysed data using Stata version 15 (StataCorp, College Station, TX, USA). Baseline characteristics of the participants were summarised using frequencies and proportions. Chi-square and Fisher’s exact tests were used to assess associations with awareness of oral PrEP. Univariable robust poisson regression was used to estimate crude prevalence ratios (PR) and 95% confidence intervals (CIs). Variables significant at p < 0.1 and those hypothesized to be associated with the outcome based on prior knowledge (i.e., condom use, age and level of education) were selected for inclusion in a multivariable robust poisson regression model. We also tested for collinearity and for the variables that were found to be highly correlated i.e. drug use and alcohol use [30] only one of these was selected and included in the final model. The final model was selected based on variables that were found to be independently associated (P < 0.05) with the outcome. After fitting the final model, adjusted prevalence ratios (aPR) and 95% CIs were obtained and reported.

### Ethical considerations

Ethical approval was obtained from the Uganda Virus Research Institute Research Ethics Committee (GC/127/18/06/658) and Uganda National Council for Science and Technology (HS2435). Participants gave written informed consent before being enrolled.

## Results

### Baseline characteristics

We screened 532 AGYW for both study participation and PrEP eligibility, 94 were not eligible for PrEP due to: HIV sero-positivity [29], not sexually active in the past 3 months [51], hepatitis B infection [10] and abnormal kidney function tests [4]. Of the rest, 153 were not eligible for the study due to: study sample size being achieved (93) and unwillingness to return for follow up procedures and other reasons (60). We therefore enrolled 285 AGYW (all PrEP eligible) and all were included in our analysis. The mean age was 19.9 (SD ± 2.24) years; 39.3% (112) were younger than 20 years old (Table 1); 54.7% had completed secondary education or greater; 57.2% were single and while 92.6% engaged in transactional sex, 21.0% reported sex work as their main occupation. The proportion of participants that were alcohol dependent based on the audit score, reported illicit drug use in the past one month, and reported frequent travelling away from home during the last 3 months were 8.8%, 84.6%, and 40.0%, respectively. Additionally, 80.0% of the participants had their first sexual encounter before the age of 18 years; 68.8% had multiple sexual partners and 73.3% reported consistent condom use in the last 3 months. The prevalence of sexually transmitted infections (STIs) i.e. chlamydia, gonorrhoea was 25.3%

**Table 1:** Socio-demographic characteristics and Chi-square analysis for demographic and behavioral correlates of oral PrEP awareness among adolescent girls and young women, Kampala, Uganda (Jan–Oct 2019)

		Heard of oral PrEP	
Variables (N = 285)	N (Col%)	n = 66 n (row%)	P-value
Age at enrollment, mean (SD)	19.9 (± 2.24)		
Age group			
14–19	112 (39.3)	26 (23.2)	
20–24	173 (60.7)	40 (23.1)	0.986
Level of education			
None/primary	129 (45.3)	31 (24.0)	
Secondary/tertiary	156 (54.7)	35 (22.4)	0.751
Marital status			
Married	84 (29.5)	14 (16.7)	
Separated/divorced	38 (13.3)	10 (26.3)	
Single (never married)	163 (57.2)	42 (25.8)	
Occupation			
Sex work	60	20 (33.3)	
Other	157 (55.1)	34 (21.7)	
No job	68 (23.9)	12 (17.7)	0.088
Alcohol consumption risk level			
Low risk drinking	196 (68.8)	38 (19.4)	
Moderate risk/ hazardous	64 (22.5)	16 (25.0)	
High risk/ Alcohol dependent	25 (8.80)	12 (48.0)	0.006
Illicit drug use in the last one month			
Yes	241 (84.6)	15 (21.2)	
No	44 (15.4)	51 (34.1)	0.062
Frequently stayed away from home in the last 3 months			
Yes	114 (40.0)	33 (28.9)	
No	171 (60.0)	33 (19.3)	0.059
Age at first sex			
9–17	228 (80.0)	58 (25.4)	
≥ 18	57 (20.0)	8 (14.0)	0.068
Number of sexual partners in the last 3 months			
1	89 (31.2)	12 (13.5)	
≥ 2	196 (68.8)	54 (27.6)	0.009
Reported consistent condom use in last 3 months			
Yes	209 (73.3)	44 (21.1)	
No	76 (26.7)	22 (28.9)	0.162
Transactional sex			
Yes	264 (92.6)	63 (23.9)	
No	21 (7.40)	3 (14.3)	0.425
STI test results (Chlamydia and Gonorrhea)			
Negative	210 (74.7)	46 (21.9)	
Positive	71 (25.3)	19 (26.8)	0.402
Contraceptive use			
Yes	158 (55.4)	35 (22.2)	
No	127 (44.6)	31 (24.4)	0.653
Willingness to use oral PrEP			
Yes	256 (89.8)	89.8	
No	29 (10.2)	10.2	
Willingness to use injectable PrEP			
Yes	255 (89.5)	89.5	
No	30 (10.5)	10.5	

### Awareness of oral and injectable PrEP

The prevalence of oral PrEP awareness was 23.2% (95% CI 18.4–28.5) while that of injectable PrEP was at 3.9% (95% CI 1.94–6.8), 6 participants had heard of both methods. Sources of oral PrEP information included peers (54.6%), health workers (42.4%), media (7.6%), other (3.0%) and community leaders (1.5%) while sources for injectable PrEP information were peers 8 (72.7%), heath workers 3 (27.3%) and media 1 (9.1%).

Oral PrEP awareness was significantly higher among participants with more than one sexual partner compared to those with only one sexual partner (27.6% vs 13.5%; p = 0.009). Additionally, alcohol dependents were more aware about PrEP compared to the low risk drinkers. (48.0% vs 19.4%; p = 0.006) (Table 1).

### Willingness to use oral and injectable PrEP

Following an explanation of the details of PrEP and its potential benefits, majority of the participants 256 (89.8%) and 255 (89.5%) were willing to use either oral or injectable PrEP respectively. The high pill burden was the main reason for non-willingness to use oral PrEP (22, 75.9%), whereas non-willingness for injectable PrEP usage was majorly explained by the fear of potential side effects (12, 40.0%).

### Factors associated with awareness of oral PrEP

In the adjusted analysis (Table 2), the prevalence of oral PrEP awareness was higher among participants who screened as alcohol dependent based on the AUDIT tool compared to those who were not (aPR 1.89, 95% CI 1.08–3.29). Also, the prevalence ratio comparing participants who reported multiple sexual partners to those with only one sexual partner was 1.84 (aPR 1.84, 95% CI 1.01–3.35). The prevalence of oral PrEP awareness was lower among participants who reported consistent condom use compared to those who did not use condoms with sexual partners in the past 3 months (aPR 0.58, 95% CI 0.37–0.91).

**Table 2:** Factors associated with awareness of oral pre-exposure prophylaxis among adolescent girls and young women, Kampala, Uganda (Jan–Oct 2019), N = 285

	uPR (95% CI)	P-value	aPR (95% CI)	P-value
Age group (age at enrolment)				
14–19	1			
20–24	0.99 (0.64–1.53)	0.986	0.93 (0.61–1.42)	0.759
Level of education				
None/primary	1			
Secondary/tertiary	0.93 (0.61–1.42)	0.751	1.02 (0.68–1.55)	0.910
Marital status				
Married	1			
Separated/divorced	1.58 (0.77–3.23)	0.212		
Single (never married)	1.54 (0.89–2.66)	0.118		
Occupation				
No job	1			
Sex work	1.88 (1.01–3.53)	0.047	1.53 (0.80–2.94)	0.194
Other^a^	1.22 (0.67–2.22)	0.500	1.15 (0.64–2.09)	0.621
Alcohol consumption risk level				
Low risk drinking	1			
Moderate/hazardous	1.28 (0.77–2.15)		1.11 (0.65–1.90)	0.677
Alcohol dependent	2.47 (1.50–4.07)	0.000	1.89 (1.08–3.29)	0.024^b^
Illicit drug use in the last one month				
No	1			
Yes	1.61 (0.99–2.59)	0.051		
Age at first sex				
9–17	1			
≥ 18	0.55 (0.27–1.09)	0.087		
Number of sexual partners in the last 3 months				
One	1			
≥ 2	2.04 (1.15–3.62)	0.015	1.84 (1.01–3.35)	0.047^b^
Frequently stayed away from home in the last 3 months				
No	1			
Yes	1.50 (0.98–2.28)	0.059		
Reported consistent condom use in last 3 months				
No	1			
Yes	0.72 (0.46–1.12)	0.156	0.58 (0.37–0.91)	0.019^b^
Contraceptive use				
No	1			
Yes	0.90 (0.59–1.38)	0.654		
STI test results (Chlamydia and Gonorrhea)				
Negative	1			
Positive	1.22 (0.76–1.94)	0.396		

*uPR* unadjusted prevalence ratio, *aPR*  adjusted prevalence-ratio, *95% CI*  95% confidence interval

^a^Hospitality, entertainment and any other job

^b^P < 0.05

Due to small numbers aware of injectable PrEP, we did not assess for factors associated with awareness of injectable PrEP, therefore the data for injectable PrEP are not presented in tables.

## Discussion

We found only one in four AGYW participants had heard about oral PrEP, and fewer than one in twenty had ever heard about injectable PrEP, a hypothetical intervention still in clinical trials at the time of this study. Peer interactions contributed more to awareness of oral and injectable PrEP when compared to other sources such as health workers and media.

We also found that most of the AGYW reported being willing to use either oral or injectable PrEP after being educated about these methods, including details on the mode of administration, frequency of use, and the fact that the drugs have been found effective in preventing HIV infection. Alcohol consumption risk, number of sexual partners and condom use were associated with Oral PrEP awareness. Our findings are consistent with other studies in sub-Saharan Africa, which have reported low oral PrEP awareness among high-risk populations [2, 18, 20, 31, 32]. This finding could explain the observed low uptake of oral PrEP in the early oral PrEP implementation phase in several communities [33]. Also within these diverse communities, there have been few awareness campaigns, which could also explain the low uptake [2, 34]. On the other hand, low awareness of oral and injectable PrEP may be attributed to the fact that these prevention methods are new and have not been fully appreciated within these communities [35]. Future HIV prevention interventions should leverage on peer to peer networks to not only increase awareness about oral PrEP and other new HIV prevention methods but to also influence uptake and adherence.

Participants with multiple sexual partners had a higher prevalence of oral PrEP awareness. Previous studies have reported similar findings [36, 37]. Since exposure to multiple partners places individuals at higher risk of acquiring different diseases including HIV, individuals involved in such practice may be more likely to search for methods to protect themselves and this could explain the higher PrEP awareness we observe within this group in the current study. Also, it is likely that those with multiple sexual partners may have deeper social and peer-based networks, perhaps increasing their access to sources of information for health including PrEP [38].

The prevalence of oral PrEP awareness was higher among non-condom users. This is similar to results reported from a study in Kenya that assessed PrEP awareness among AGYW [39]. One reason for this would be misconceptions about condom use that drives the search for alternative methods of protection other than condoms within this group of individuals [40, 41]. Furthermore, it is likely that non-condom users just do not like condoms or cannot use condoms because sexual partners may control condom use in a relationship [42, 43]. Non condom users may therefore be driven by the need to protect themselves from HIV to search for and learn about alternative novel methods such as oral PrEP. Oral PrEP has been identified as a more user friendly and user-controlled method for AGYW and likely a suitable alternative for those who may not have skills to negotiate condom use with sexual partners [44]. Also, those who do not use condoms are likely to be more aware of PrEP in this population with a high prevalence of transactional sex. In our study setting, female sex workers are often offered higher fees by their clients in return for condomless sex [45]. This might explain why some participants prefer not to use condoms. In such circumstances, the need to protect themselves could lead them to search for or learn about more discrete methods that are not partner controlled e.g. oral PrEP [46, 47].

While our study suggests higher PrEP awareness among AGYW with alcohol dependence, other studies have reported PrEP awareness to be lower among persons with alcohol dependence compared to low risk alcohol consumers [36]. The majority of volunteers (> 90%) in our study engaged in transactional sex; alcohol consumption in this context occurs within sex work venues such as bars and nightclubs often as a way of coping with sex work [48]. It is possible that during the national oral PrEP roll out, the Ministry of Health and other stakeholders prioritized oral PrEP educational interventions in locations where key populations congregated, e.g., bars and nightclubs [49].

Peer influence was a key contextual factor contributing to awareness of oral PrEP. Peers with whom AGYW interacted were also potential sources of information about oral PrEP. Future HIV prevention interventions should leverage on peer to peer networks to not only increase awareness about oral PrEP but to influence uptake and adherence.

Alcohol dependent individuals tended to spend a long time away from their homes, which exposes them to meeting new people who may become sexual partners [50]. This is consistent with results reported by several studies that have indicated a correlation between alcohol dependency and high-risk sexual behavior among young people [51, 52] and older populations at risk [53, 54]. If aware of such risk, persons dependent upon alcohol may be more aware of the available methods that may protect them from contracting HIV.

Our study has three main limitations. First, this study focused on AGYW at risk within peri-urban areas and results may not be generalizable to at-risk AGYW found in rural areas and other regions of sub-Saharan Africa. Second, it was a cross-sectional study and so we were only able to assess the association between factors and PrEP awareness but could not establish causal directions. Last, the questionnaire assessed the source of PrEP awareness but did not collect information on whether the participant sought this knowledge for themselves or received it passively hence speculating on the reasons for the noted associations. Despite these limitations, our study was the first in Uganda to show that oral PrEP awareness remains low among this AGYW at high risk of HIV infection.

## Conclusion

Promoting awareness for new effective HIV prevention tools among high risk AGYW is critical and should be conducted in a timely manner. In addition, this needs to be scaled up to potentially impact demand, uptake and adherence.

## Data Availability

The datasets used and analyzed during the current study are available from the corresponding author on reasonable request.

## Abbreviations

AGYW: Adolescent girls and young women
PrEP: Pre-exposure prophylaxis
AUDIT: Alcohol use disorders identification test
CI: Confidence interval
PR: Prevalence ratio
WHO: World Health Organization
HIV: Human immunodeficiency virus
FSW: Female sex worker
HCT: HIV counselling and testing
STI: Sexually transmitted disease
GHWP: Good Health for Women Project
UNCST: Uganda National Council for Science and Technology
UVRI-REC: Uganda Virus Research Institute Research Ethics Committee
MoH: Ministry of Health
